# Prevalence and predictors of Lymphogranuloma venereum in a high risk population attending a STD outpatients clinic in Italy

**DOI:** 10.1186/1756-0500-7-225

**Published:** 2014-04-09

**Authors:** Claudio Foschi, Antonella Marangoni, Antonietta D’Antuono, Paola Nardini, Monica Compri, Sara Bellavista, Andrea Filippini, Maria Letizia Bacchi Reggiani, Roberto Cevenini

**Affiliations:** 1 Microbiology, DIMES, University of Bologna, St. Orsola Hospital, Via Massarenti, 9, 40138 Bologna, Italy; 2 Dermatology, DIMES, University of Bologna, St. Orsola Hospital, Via Massarenti, 9, 40138 Bologna, Italy; 3 Cardiology, DIMES, University of Bologna, St. Orsola Hospital, Via Massarenti, 9, 40138 Bologna, Italy

**Keywords:** LGV, MSM, *Chlamydia trachomatis*, NAATs

## Abstract

**Background:**

We evaluated LGV prevalence and predictors in a high risk population attending a STI Outpatients Clinic in the North of Italy.

**Methods:**

A total of 108 patients (99 MSM and 9 women), with a history of unsafe anal sexual intercourses, were enrolled. Anorectal swabs and urine samples were tested for *Chlamydia trachomatis* (CT) DNA detection by Versant CT/GC DNA 1.0 Assay (Siemens Healthcare Diagnostics Terrytown, USA). RFLP analysis was used for CT molecular typing.

**Results:**

L2 CT genotype was identified in 13/108 (12%) rectal swabs. All LGV cases were from MSM, declaring high-risk sexual behaviour and complaining anorectal symptoms. Patients first attending the STI Outpatient Clinic received a significant earlier LGV diagnosis than those first seeking care from general practitioners or gastroenterologists (*P* = 0.0046).

LGV prevalence and characteristics found in our population are in agreement with international reports. Statistical analysis showed that LGV positive patients were older (*P* = 0.0008) and presented more STIs *(P* = 0.0023) than LGV negative ones, in particular due to syphilis *(P* < 0.001), HIV (*P* < 0.001) and HBV (*P* = 0.001).

Multivariate logistic regression analysis revealed that HIV and syphilis infections are strong risk factors for LGV presence (respectively, *P* = 0.001 and *P* = 0.010).

**Conclusions:**

Even if our results do not provide sufficient evidence to recommend routine screening of anorectal swabs in high-risk population, they strongly suggest to perform CT NAAT tests and genotyping on rectal specimens in presence of ulcerative proctitis in HIV and/or syphilis-positive MSM. In this context, CT DNA detection by Versant CT/GC DNA 1.0 Assay, followed by RFLP analysis for molecular typing demonstrated to be an excellent diagnostic algorithm for LGV identification.

## Background

Lymphogranuloma venereum (LGV) is a systemic sexually transmitted infection caused by *Chlamydia trachomatis* (CT) serovars L1- L3 [1].

In 2003, a new outbreak of LGV proctitis was described in the Netherlands, mainly in men who have sex with men (MSM) [2], leading to an increase awareness for this disease throughout Europe.

The first Italian LGV case was observed in Milan in 2006 and only few cases of LGV proctitis have been described in Italy [3] so far.

In this study we assess LGV prevalence and predictors in a high-risk population attending a STI Outpatients Clinic of a University Hospital in the North of Italy.

## Methods

### Study population

From January 2012 to April 2013, all the patients attending the STI Outpatients Clinic of St. Orsola University Hospital of Bologna and reporting unsafe anal sexual intercourses have been asked to carry out a clinical examination. An anorectal swab, a pharyngeal swab (if reporting oral sex intercourses) and an urine sample were collected from each patient for DNA detection of CT and *Neisseria gonorrhoeae* (GC).

Microbiological investigations for the main STDs (HIV, HCV, HBV and syphilis) and a serological screening for anti-*Chlamydia* antibodies by immunoenzimatic assays (Chlamydia IgG and Chlamydia IgA, Virion/Serion GmbH, Wurzbug, Germany) were performed in all patients. Diagnosis of genital warts was made by visual inspection. Furthermore, personal data and information about urogenital and rectal disorders, sexual behaviour, number of sexual partners in the last 6 months and history of previous STIs were recorded from each patient. Three months after antibiotic treatment for LGV, patients were re-evaluated.

A written consent was obtained by all the patients and the study protocol was reviewed by the Ethics committee of St. Orsola Hospital.

### Diagnosis of CT infections and genotyping

Urine specimens, anorectal and pharyngeal swabs were processed by Versant CT/GC DNA 1.0 Assay (Siemens Healthcare Diagnostics, Terrytown, USA), a Real-Time PCR test simultaneously detecting the presence of CT and/or GC DNA [4]. In case of a CT positive result, molecular genotyping, based on *omp1* gene semi-nested PCR, followed by RFLP analysis was performed as previously described [5-7]. GC reactive results were confirmed by in-house PCR assay targeting *porA* pseudogene [8].

#### Statistical analysis

Analyses of the differences between the groups were performed with χ2 test. Univariate and multiple logistic regression analyses were performed to evaluate the influence of the different variables on the outcomes. A *P* value <0.05 was considered significant. Statistical tests were performed using SPSS 13.0 for Windows computer software (SPSS, Chicago, Illinois).

## Results

### Patients characteristics

During the study period, a total of 108 patients met our admission criteria. In particular, 99 MSM (median age: 34.9; range 18–64 years) and 9 heterosexual women (median age: 35.8; range 19–52 years). Three of the women and 29 of the MSM complained about various rectal symptoms.

### Diagnosis of CT infections and genotyping

Nineteen rectal swabs resulted positive only for CT and 10 only for GC, whereas 4 were simultaneously scored positive for CT and GC. Thanks to molecular genotyping, in 10 cases we found non-LGV serovars CT (6 E, 3D, 1 J), while in 13 cases L2 serovar was identified, coming to the final diagnosis of LGV proctitis. The total prevalence of LGV infection was 12% (13/108).

Four urine samples were positive for non-LGV serovars CT, whereas 12 pharyngeal swabs and three urine specimens were positive for GC. No urine samples nor pharyngeal swabs were found positive for LGV-serovars CT. Finally, it is noteworthy to underline that in the high risk population of this study 38.3% of MSM (38/99) and 37.5% of the women (3/8) had at least one specimen scored CT or GC positive.

**Figure 1:**
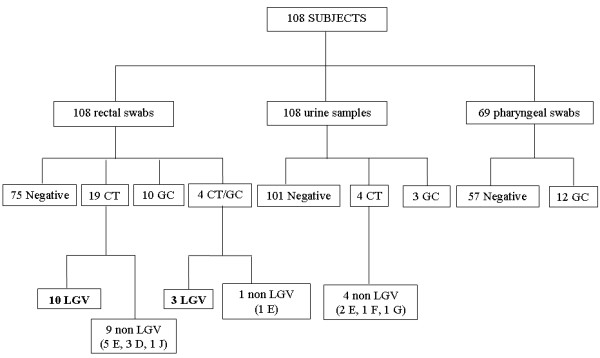
**CT and GC testing results.** Flowchart of testing of 108 high risk subjects for CT and GC by Versant CT/GC DNA 1.0 Assay. Results obtained by commercial NAAT were confirmed by *omp1* and *porA* in-house PCR assays.

Detailed results are shown in Figure 1.

#### Clinical findings, risk factors and outcome of LGV cases

Based on rectal swab findings, patients with positive LGV results were defined as LGV-positive, while all the others (negative, non-LGV CT or GC cases) as LGV-negative.

All LGV cases were detected in MSM reporting unsafe receptive anal intercourses in the last 6 months.

In Table 1 statistical differences between LGV positive and LGV negative groups and logistic regression analysis to determine risk factors for LGV presence are presented in Table 2.

**Table 1:** Statistical analysis of the subjects by their LGV status

	**LGV negative**	**LGV positive**	***P*** **value**
	**N = 95**	**N = 13**	
**Reported symptoms % (n)**	20% (19)	100% (13)	<0.001
**MSM %(n)**	91.6% (87)	100% (13)	0.601
**Mean age (±SD), (range)**	34 years (±9.5), (18–64)	43 years (±6.3), (27–53)	0.0008
**Mean anti-Chlamydia IgG AU/ml, (range)**^**£**^	11.8 (4–38)	60.4 (19–86)	<0.001
**Presence of coinfections**^**†**^**% (n)**	47.3% (45)	92.3% (12)	0.0023
**HIV % (n)**	10.5% (10)	69.2% (9)	<0.001
**Syphilis % (n)**	16.8% (16)	69.2% (9)	<0.001
Gonorrhoea* % (n)	17.8% (17)	23.0% (3)	0.6519
Non-L serovar CT* % (n)	13.7% (13)	0% (0)	0.155
**HBV % (n)**	3.2% (3)	30.8% (4)	0.001
**HCV % (n)**	1.1% (1)	7.7% (1)	0.570
**HPV warts % (n)**	10.5% (10)	30.8% (4)	0.110

† A patient was considered coinfected when at least one of the following infections was present: HIV, gonorrhoea, non-L CT, HBV, HCV, HPV warts.

*A patient was considered gonorrhoea or non-L CT positive when at least one of the site tested (pharyngeal swab and/or rectal swab and/or urine) resulted positive.

^£^ Normal values: IgG <15 Arbitrary Units/ml.

**Table 2:** Univariate and multivariate logistic regression analysis for LGV risk factors

	**Univariate analysis**		**Multivariate analysis**	
	**OR (CI 95%)**	***P*** **value**	**OR (CI 95%)**	***P*** **value**
**Age (years)**	1.11 (1.03-1.19)	0.003		
**Presence of coinfections**	13.33 (1.66-106.66)	0.014		
**HIV**	19.12 (4.96-73.61)	<0.001	12.64 (3.00-53.25)	0.001
**Syphilis**	11.10 (3.04-40.54)	<0.001	6.72 (1.58-28.45)	0.010
**Gonorrhoea**	1.37 (0.34-5.54)	0.653		
**HBV**	13.62 (2.62-70.68)	0.002		
**HCV**	7.8 (0.45-133.56)	0.155		
**HPV warts**	3.77 (0.98-14.54)	0.053		

All patients suffering from LGV proctitis were symptomatic, complaining about anal pain (13/13), anal discharge (11/13), change in bowel habit (7/13), tenesmus (9/13) and inguinal adenopathy (5/13). In contrast, patients with non-LGV CT or GC proctitis were asymptomatic or complained about slight symptoms. In particular, only 30% CT and 40% GC positive patients were symptomatic respectively, in contrast to 100% LGV positive patients (*P* < 0.001).

When considering the mean age of LGV positive patients, we noticed a significant difference between LGV and non-LGV group (43 years vs. 34 years; *P* = 0.0008), as well as comparing LGV cases with non LGV-CT positive patients (43 years vs. 36 years; *P* = 0.04).

All LGV positive patients showed altered anti-*Chlamydia* IgG values, significantly higher than LGV-negative subjects (*P* < 0.001). Moreover, it is interesting to add that patients infected by non-LGV CT genotypes showed similar results to CT-negative patients (*P* = 0.102). Anti-*Chlamydia* IgA values were negative or only slight increased with no significant differences between studied groups.

All LGV positive patients but one suffered from other STIs; in particular, 9 were HIV-positive. Six of them were under HAART-therapy with a well control of the infection (viral load <1000 copies/ml; CD4 > 500 cells/ml).

When evaluating the distribution of other STIs in our population, we noticed that LGV positive patients presented more coinfections than LGV negative subjects (*P* = 0.001).

Finally, considering LGV cases, patients first attending the STI Outpatients Clinic (4/13) received an earlier diagnosis compared to patients who first sought help from general practitioners or gastroenterologists. In particular, in the former group, time taken for LGV diagnosis was 2 weeks (range: 10–18 days), in contrast with the median of 8 weeks (4–92 weeks) of the latter group (*P* =0.0046). First diagnostic hypothesis included inflammatory bowel diseases (IBD), enteritis and other STIs with rectal localization such as syphilis or gonorrhoea.

LGV positive patients were treated with doxycycline 100 mg twice a day for 3 weeks. They completely recovered after antibiotic therapy, regardless of their HIV status, HIV viral load or CD4 cell count.

Three months after treatment, rectal swabs and urine samples were negative both for CT and GC nucleic acids in all patients but one. This patient had a positive result for GC and a non-LGV serovar CT in his rectal swab, with recurrence of mild anal symptoms.

As expected, at follow-up visit anti-*Chlamydia* IgG antibodies values were similar to those found at the moment of LGV diagnosis.

## Discussion

In the last decade, a new outbreak of LGV infection with rectal localization has been described in Europe, mainly in MSM [2]. International data about prevalence of LGV infection show considerable variability, depending on the characteristics of studied groups, with highest levels on high-risk selected population [9,10].

In our study, we found 13 cases of rectal LGV in a high-risk population and prevalence of LGV proctitis was 12%.

As shown in our investigation, LGV proctitis is usually symptomatic and it occurs as a moderate or severe ulcerative proctitis in more than 90% of cases [11], while non-LGV CT proctitis remains clinically silent in many cases [12].

In agreement with international literature [13-15], we observed that all LGV cases were diagnosed in MSM and that LGV positive patients were significantly older than LGV-negative ones (*P* = 0.0008).

Although patients reported active and passive anal intercourses, a LGV urethral infection has not been identified in our population. This is in concordance with previously reported studies [2,9] even if recently de Vrieze *et al.* found some cases of concurrent urethral LGV in a group of MSM with anorectal infection [16].

As observed in our cases, LGV proctitis can be easily misdiagnosed at the first investigations due to the unawareness of general practitioners and shame of patients in seeking care [11]. The most common misdiagnosis concerns IBD, since both endoscopic appearance of rectal mucosa and histological examination show similar characteristics [17].

High-risk sexual behaviours, including high number of sexual partners, unsafe intercourses, fisting and sex toys sharing are reported by most of LGV infected patients [18,19]. Common risk behaviours and LGV ulcerative lesions can both explain the high prevalence of other STIs found in LGV patients.

Our data highlighted a significant difference between LGV-positive and LGV-negative subjects regarding coinfections due to other STIs (*P* = 0.0023); in particular among LGV patients higher rates of HIV (*P* < 0.001), syphilis (*P* < 0.001) and HBV (P = 0.001) infections were found. Logistic regression analysis in our population showed that HIV and syphilis represent the strongest risk factors for LGV infection (P < 0.001).

A strong association between anorectal LGV and HIV seropositivity has been already well documented [18], with rate of co-infection reaching 60-100% in MSM population [20].

It has been supposed that HIV-related immunodeficiency could delay LGV clearance [21]. Nevertheless, antibiotic treatment for LGV with doxycycline for 21 days appears to be equally effective both in HIV-negative and HIV positive patients [14].

As already observed [18], we confirm that LGV clinical presentation was not influenced by the use of HAART therapy and LGV outcome did not depend on HIV viral load or CD4 cell count.

Laboratory investigations have a crucial role in LGV diagnosis, but the quest for a reliable and simple method to discriminate between LGV and non-LGV CT infections is still going on. Both serological and nucleic acid amplification techniques (NAATs) indeed present critical issues.

*Chlamydia* serology can support LGV diagnosis in the appropriate clinical context, but the diagnostic utility of serological methods other than complement fixation and microimmunofluorescence procedures has not been well established yet [22,23]. In this study, we found a significant elevation of anti-*Chlamydia* IgG by an immunoenzimatic assay in LGV-positive patients, suggesting a potential role of IgG testing for LGV diagnosis.

On the other hand, NAATs have not been FDA cleared or CE marked yet on rectal specimens.

For that reason, many laboratories have performed their own validation studies in order to provide results for clinical management [22]. Moreover, since commercial NAATs can not differentiate LGV from non-LGV serovars, further investigations for CT molecular typing are needed. In the absence of specific LGV diagnostic testing, patients with a clinical syndrome consistent with LGV, should be treated with LGV regimen (doxycycline 100 mg orally twice a day for 21 days) [15,23].

In our experience, even if Versant CT/GC DNA 1.0 Assay has been not licensed for extragenital use, it showed good performances in CT/GC DNA detection on rectal and pharyngeal swabs. In addition, RFLP analysis of CT positive samples seems to be a reliable, inexpensive and time-saving method for CT genotyping.

It follows that our suggested algorithm (i.e. Versant CT/GC DNA 1.0 Assay and RLFP analysis of CT positive samples) could be an excellent choice for laboratory LGV diagnosis.

Even if our results do not provide sufficient evidence to recommend routine screening of anorectal swabs in MSM population, they confirm, in agreement with European guidelines, the indication to perform CT NAAT tests and genotyping on rectal specimens in presence of ulcerative proctitis or in sexual contacts of LGV positive people [9,24].

In particular, our results suggest to test for rectal LGV all MSM complaining of anorectal symptoms, especially if other STIs (HIV and/or syphilis) are identified. Moreover, these subjects should be presumptively treated for LGV infection until CT molecular genotyping is known.

## Conclusions

On the strength of our epidemiological and statistical results, testing for LGV infection should be recommended to all HIV and/or syphilis-positive MSM complaining of anorectal symptoms. For this purpose, CT DNA detection by Versant CT/GC DNA 1.0 Assay, followed by RFLP analysis for molecular typing demonstrated to be an excellent diagnostic algorithm for LGV identification.

## Competing interests

The authors declare that they have no competing interests.

## Authors’ contributions

AM, AD, RC conceived and designed the study; CF, PN, MC, SB, AF performed the clinical visit and sample testing; CF, AM, LBR analyzed the data; CF, AM, RC wrote the paper; all authors read and approved the final manuscript.
